# Promising Therapeutic Impact of Immune Checkpoint Inhibitors in Type II Endometrial Cancer Patients with Deficient Mismatch Repair Status

**DOI:** 10.3390/healthcare11081073

**Published:** 2023-04-09

**Authors:** Kiyoka Sawada, Kentaro Nakayama, Sultana Razia, Hitomi Yamashita, Tomoka Ishibashi, Masako Ishikawa, Kosuke Kanno, Seiya Sato, Satoru Nakayama, Yoshiro Otsuki, Satoru Kyo

**Affiliations:** 1 Department of Obstetrics and Gynecology, Shimane University School of Medicine, Izumo 6938501, Japan; kiyoka-s@med.shimane-u.ac.jp (K.S.); raeedahmed@yahoo.com (S.R.); memedasudasu1103@gmail.com (H.Y.); tomoka@med.shimane-u.ac.jp (T.I.); m-ishi@med.shimane-u.ac.jp (M.I.); kanno39@med.shimane-u.ac.jp (K.K.); sato_seiya9534@yahoo.co.jp (S.S.);; 2 Department of Obstetrics and Gynecology, Seirei Hamamatsu Hospital, Hamamatsu 4308558, Japan; satoru@sis.seirei.or.jp; 3 Department of Pathology, Seirei Hamamatsu Hospital, Hamamatsu 4308558, Japan; otsuki@sis.seirei.or.jp

**Keywords:** endometrial cancer, type II, immune checkpoint inhibitors, deficient mismatch repair system

## Abstract

Type II endometrial cancer (EC) is responsible for most endometrial cancer-related deaths due to its aggressive nature, late-stage detection, and high tolerance to standard therapies. Thus, novel treatment strategies for type II EC are imperative. For patients with mismatch repair-deficient (dMMR) tumors, immunotherapy with immune checkpoint inhibitors represents a promising therapeutic strategy. However, the prevalence of dMMR tumors in type II EC patients remains unclear. In this study, using immunohistochemistry, we evaluated the expression of mismatch repair (MMR) proteins, tumor-infiltrating lymphocytes (CD8+), and immune checkpoint molecules (PD-L1) in 60 patients with type II EC (16, 5, 17, and 22 were endometrioid G3, serous, de-differentiated, and carcinosarcoma cases, respectively) to investigate the therapeutic effect of immune checkpoint inhibitors. Approximately 24 cases (40%) had a loss of MMR protein expression. The positivity rate of CD8+ (*p* = 0.0072) and PD-L1 (*p* = 0.0061) expression was significantly associated with the dMMR group. These results suggest immune checkpoint inhibitors (anti-PD-L1/PD-1 antibodies) could effectively treat type II EC with dMMR. The presence of dMMR might be a biomarker for a positive response to PD-1/PD-L1 immunotherapy in type II EC.

## 1. Introduction

Cancer is responsible for one in six deaths worldwide and is thereby the second leading cause of mortality [1]. An estimated 3.6 million people, mostly women, died from cancer in 2012, while 6.6 million new cases were reported. Among gynecological cancers, endometrial carcinoma (EC) is the most prevalent and ranks fourth among cancers in women, accounting for 97,000 deaths globally in 2020 [2]. Women between the ages of 60 and 75 years develop EC during or after menopause. Most ECs (97%) are epithelial lesions arising from the lining of the uterus [3]. In 1983, Bokman classified ECs into two types based on the histopathology of the tumors [4]. Type I ECs are generally well to moderately differentiated low-grade endometrioid tumors and account for 80–85% of all ECs [5]. Type I tumors develop in an estrogenic environment with a good prognosis having a 5-year survival rate of >80% due to their indolent clinical course and early-stage detection [6]. In contrast, type II ECs are high-grade by definition and represent approximately 15–20% of all ECs. They are generally poorly differentiated, without steroid receptors, and of a nonendometrioid histological subtype. Type II ECs have a poor prognosis as they are unresponsive to antiestrogen therapy and have a high recurrence rate. These tumors often arise within the atrophic endometrium [7]. Although type II tumors occur less frequently than type I tumors, the former are associated with a higher mortality rate. Hamilton et al. [8] reviewed the Surveillance, Epidemiology, and End Results (SEER) data and found that 11% of ECs (G3) were type II; this subtype was responsible for 47% of deaths in their analysis. In addition, the 5-year overall survival (OS) rates for type II tumors were considerably lower than those for type I tumors after adjustment for stage [9]. Therefore, novel treatments for type II EC are urgently required.

Recently, molecular-targeted drugs have gained attention due to the development of genomic medicine, and immunotherapy has revamped cancer therapeutic paradigm. The immune system plays an essential role in the recognition and arrest of the development of cancer [10]. The PD-1 surface receptor is expressed on some tumor cells and, by attaching to PD-L1 on cytotoxic lymphocytes, it suppresses lymphocyte activation and maintains immune evasion. Immune checkpoint inhibitors (ICIs) remove the “brakes” from the immunological system (primary T cells and dendritic cells), allowing an immune response to and elimination of cancer cells instead of targeting cancer cells, which is the mechanism of chemotherapy, targeted therapy, and radiation therapy. ICI therapy has revolutionized the management of some metastatic and advanced cancers and changed their prognosis and treatment protocols. Among the ICIs, PD-1/PD-L1 and CTLA-4 inhibitors have demonstrated promising therapeutic results. Some of them have received approval for use in certain cancer therapies, while others are still being clinically tested. Anti-PD-1/PD-L1 immunotherapy has proved incredibly effective in the treatment of certain cancers such as melanoma, non-small cell lung cancer, metastatic colorectal cancer, renal cell carcinoma, bladder cancer, head and neck squamous cell carcinoma, classical Hodgkin’s lymphoma, and Merkel cell carcinoma [11,12,13,14,15,16,17,18,19,20,21,22,23]. In patients with non-small cell lung cancer or metastatic melanoma, only 15.2–20% achieved an objective response with this immunotherapy. Another study stated that anti-PD-1/PD-L1 antibodies were ineffective in most patients, and response efficiencies were reported to be 20–30% when patients with various cancer types were treated with PD-1 antibodies [15,24,25]. Furthermore, only a small percentage of individuals respond well to immunotherapy; therefore, specific biomarkers are urgently needed to distinguish the sensitive patients and predict therapeutic responses.

The mismatch repair (MMR) pathway plays a crucial role in correcting DNA replication errors in normal and cancerous cells. Humans have at least seven MMR proteins, although only four of them are clinically significant in terms of the mechanism of human cancer development; these include MLH1, MSH2, MSH6, and PMS2 [26]. These four proteins are organized as heterodimers, in which MLH1 connects with PMS2 and MSH2 associates with MSH6. The MSH2/MSH6 pairing acts as an endonuclease, whereas the MLH1/PMS2 pairing detects mismatched nucleotide base pairs and starts repair. These proteins are encoded by their corresponding genes (MMR genes): *MLH1*, *MSH2*, *MSH6,* and *PMS2*. Loss-of-function of one or more MMR proteins (deficient MMR; dMMR) leads to impaired DNA repair capability, and the loss-of-function of these gene products results in dMMR that is connected to a condition known as microsatellite instability (MSI), which involves changes in the size of microsatellites. This results in the accumulation of spontaneous genetic mutations across the genome leading to an increased risk of developing neoplasia. Therefore, dMMR is the most prevalent cause of hereditary EC and is linked to an elevated risk of numerous forms of cancer. In general, dMMR is equivalent to MSI-High (MSI-H) [27]. Recently it has been found that dMMR tumors lead to abundant mutation-derived neoantigens, which stimulate antitumor immune responses. In addition, new research indicates that dMMR tumors have higher levels of cytosolic DNA, which activates the cGAS-STING pathway and results in an interferon-mediated immune response. [28]. Several studies have demonstrated that dMMR is a positive predictor for response to ICIs [29]. Jin et al. identified frequent and durable responses that improved patient survival in metastatic colorectal cancer with dMMR when treated with an anti-PD-1 antibody (pembrolizumab) [30]. It is significant to note that among 30 human cancer types, EC was recently revealed to have the highest prevalence of MSI [31]; approximately 30% of primary ECs were MSI-H, and 13% to 30% of recurrent ECs were MSI-H or dMMR [31,32,33,34,35]. ICIs may therefore be a good option for the treatment of both primary and recurrent ECs. In December 2018, Japan approved pembrolizumab for treating advanced or recurrent MSI-H tumors following conventional chemotherapy. Therefore, MSI-H tumors are thought to have a favorable response to ICIs.

EC is a gynecological tumor frequently showing MMR deficiency (25–30%) [32,36,37]. Tumor-infiltrating lymphocytes (CD8+) and immune checkpoint molecules PD-L1/PD-1 expression were significantly higher in the dMMR group than in the MMR-proficient (pMMR), implying that ICIs could be effective in type I EC with dMMR [38]. This study has been further modified to evaluate whether dMMR could be a biomarker for ICI response in patients with type II EC. The prevalence of dMMR tumors in type II EC remains unclear. Hence, the study aimed to evaluate the relationship between MMR status, lymphocyte infiltration into the tumor, and the expression of immune checkpoint molecules by histological staining in type II EC. We believe that our study is the first to provide an overview of the MMR status in type II EC.

## 2. Materials and Methods

### 2.1. Tissue Samples

Samples were obtained from 60 patients with type II EC treated between January 2006 and January 2020 in the Department of Obstetrics and Gynecology at the Shimane University Hospital and Seirei Hamamatsu General Hospital. Among the 60 patients, 16, 5, 17, and 22 were endometrioid G3, serous, de-differentiated, and carcinosarcoma patients, respectively. Tissue collection and clinicopathological features of 17 de-differentiated endometrial carcinoma samples have been previously described [39]. All patients underwent total hysterectomy, bilateral salpingo-oophorectomy with or without pelvic, or para-aortic lymphadenectomy followed by carboplatin and taxane chemotherapy (paclitaxel = 175 mg/m^2^ and carboplatin area under the curve = 5 mg/m^2^). The collected samples were formalin-fixed, paraffin-embedded tissue blocks. The tumor components were collected macroscopically, based on the conventional morphological examinations of hematoxylin and eosin-stained sections. The grading of type II EC was performed according to the surgical staging system of the International Federation of Gynecology and Obstetrics (FIGO 2008) [40]. In addition, all tumors were histologically classified according to the World Health Organization criteria. Clinical information was obtained, retrospectively, from electronic medical records. The follow-up period ranged from 5 to 156 months, with a mean of 58 months. The acquisition of tumor tissue was approved by the Shimane University Institutional Review Board (IRB No. 20070305-1 and No. 20070305-2).

### 2.2. Immunohistochemistry

Expression of MMR proteins (MLH1, PMS2, MSH2, and MSH6), CD8+ lymphocyte infiltration into the tumor, and immune checkpoint molecule PD-L1 expression were evaluated by immunohistochemistry (IHC). Formalin-fixed, paraffin-embedded sections were sectioned at four-micrometers, dewaxed in xylene, and hydrated using graded alcohol. Antigen retrieval was performed by autoclaving at 121 °C using sodium citrate buffer and subsequently, slides were incubated for 30 min in phosphate-buffered saline (PBS) with 3% H_2_O_2_ and rinsed thrice with PBS. The slides were then incubated overnight at 4 °C with antibodies against MutS Protein Homolog 2 (1:50; Dako, Santa Clara, CA, United States), MutS Protein Homolog 6 (1:50; Dako), Postmeiotic Segregation Increased 2 (1:40; Dako), MutL Protein Homolog 1 (1:50; Dako), CD8 (1:100; Roche, Basel, Switzerland), and PD-L1 (1:100, ab205921, Abcam, Cambridge, UK). The HRP-conjugated secondary antibodies were added to the sections on the slides and incubated in a humidified chamber at room temperature for 30 min before visualization with DAB substrate solution. The slides were then dehydrated with graded alcohol, cleared with xylene, and coverslips mounted using mounting solution. The color of the antibody staining in the tissue sections was observed under light microscopy. The intensities of the nuclear staining were recorded. The adjacent normal tissue provided an internal positive control, and negative controls generated without the addition of primary antibody showed low background staining. A tumor was considered dMMR if at least one of the four MMR proteins was negative; all other cases were considered pMMR. The expression of CD8+ was assessed as follows: 0, undetectable; 1+, low density; 2+, moderate density; and 3+, high density. The percentage of labeled cells was distributed as follows: 0, no positive tumor cells; 1, 0–30% of tumor cells were positive; 2, 30–60% of tumor cells were positive; and 3, ≥60% of tumor cells were positive. Cases that were 2+ or 3+ were considered positive in our analysis for CD8. For PD-L1, tumor cells with ≥5% positive membranous or cytoplasmic staining were considered positive. Without prior knowledge of the clinicopathological factors, two researchers (K.S. and K.N.) evaluated the samples using a light microscope. Although we did not conduct genomic MSI analysis in this study, all 24 patients assessed to have dMMR according to IHC were considered to have MSI-H as per the results of our previous study [38,39].

### 2.3. dMMR Considered as MSI-H

In our previous study, with the aid of IHC, MMR deficiency was identified in 9 (52.9%) of the 17 patients with de-differentiated EC, and 42 (28.2%) of the 149 patients with EC. We analyzed genomic MSI in 3 cases out of the 9 patients with de-differentiated EC, and 12 cases out of the 42 patients with EC evaluated as dMMR through IHC and observed that they were all MSI-H based on MSI analysis [38,39]. Therefore, we believe that assessment of dMMR by IHC is equivalent to MSI-H.

### 2.4. Statistical Analysis

Statistical analyses were performed using the SPSS 24.0 software (IBM Corporation, Armonk, NY, USA). The chi-square test was used to analyze the association between the status of MMR and expressions of CD8+ and PD-L1. The progression-free survival (PFS) and OS were compared using Kaplan-Meier curves and log-rank tests. PFS was calculated from the first treatment date to the recurrence date or last follow-up date, whereas OS was defined using the date of diagnosis to the death date or last follow-up date. A *p* value of <0.05 was considered statistically significant.

## 3. Results

### 3.1. Patients’ Clinicopathological Characteristics

The clinicopathological features of the 60 type II EC patients are summarized in Table 1. FIGO stages I and II were identified in 25 patients, while stages III and IV were identified in 35 patients. The IHC results of MMR status was correlated to clinicopathologic variables. We found no significant association between dMMR and age *(p* = 0.093), histological grade (*p* = 0.263), FIGO stage (*p* = 0.593), pelvic lymph node metastasis (*p* = 0.093), or depth of myometrial invasion (*p* = 0.733).

### 3.2. IHC Findings

**Figure 1:**
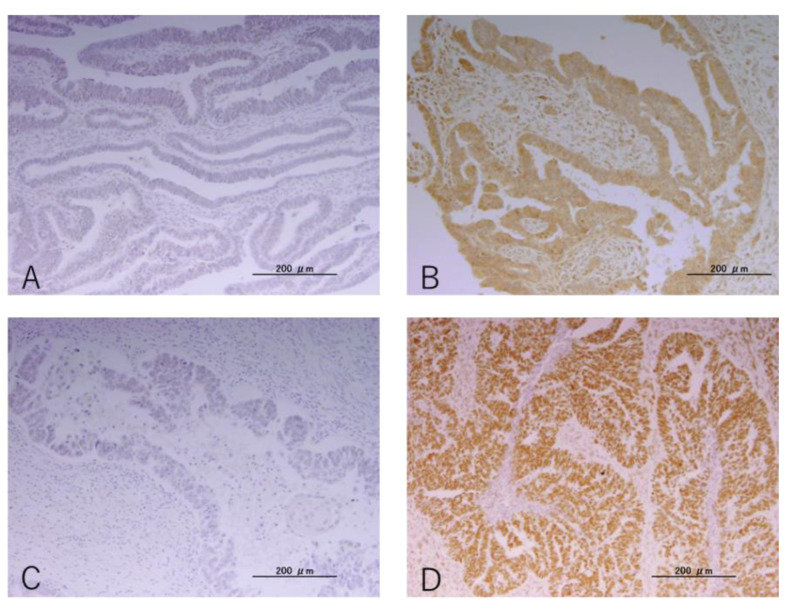

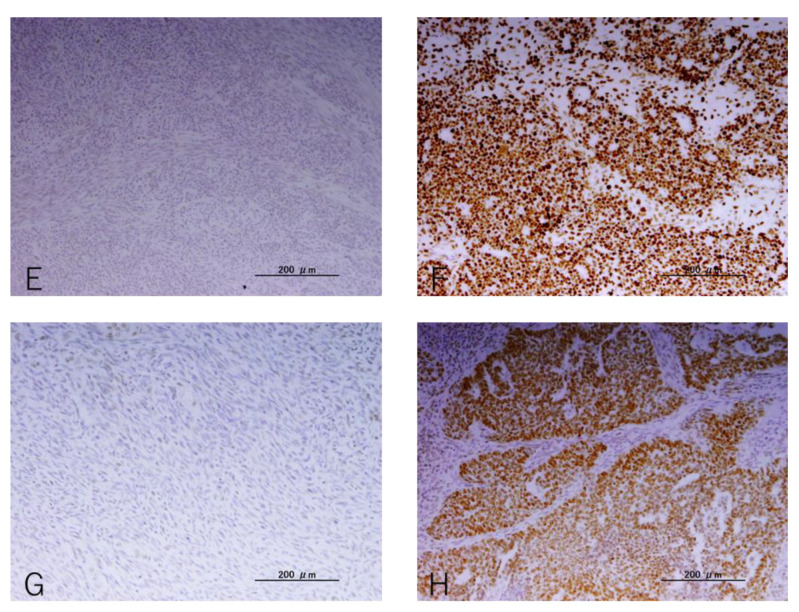
Representative images of immunostaining of MMR proteins in type II EC. (**A**) There is no expression of MLH1. (**B**) There is positive expression of MLH1. (**C**) There is no expression of MSH2. (**D**) There is positive expression of MSH2. (**E**) There is no expression of MSH6. (**F**) There is positive expression of MSH6. (**G**) There is no expression of PMS2. (**H**) There is positive expression of PMS2.

In this study, 24/60 (40%) patients were dMMR (MLH1 loss, 10 cases; PMS2 loss, 12 cases; MSH2 loss, 11 cases; and MSH6 loss, 5 cases). Only one MMR heterodimer was affected; either MLH1/PMS2 was affected in 6 of 24 (25%) cases or MSH2/MSH6 was affected in 12 of 24 (50%) cases. Simultaneous loss of immunoexpression in the protein of both heterodimers occurred in 8 (33.3%; MLH1/PMS2) and 2 (8.3%; MSH2/MSH6) cases, respectively. Interestingly, concurrent loss of MLH1/PMS2 and MSH2, PMS2 and MSH2, and MLH1 and MSH2 was observed in 1 case, 2 cases, and 1 case, respectively. Figure 1 shows the immunohistochemical findings in representative images that were positive and negative for MLH1, MSH2, MSH6, and PMS2. We used the IHC results of the de-differentiated carcinoma, which we previously reported [38]. Immunoexpression of MMR proteins in type II EC patients with dMMR is summarized in Table S1.

### 3.3. Relationship between the Status of MMR and CD8 or PD-L1 Expression

**Figure 2:**
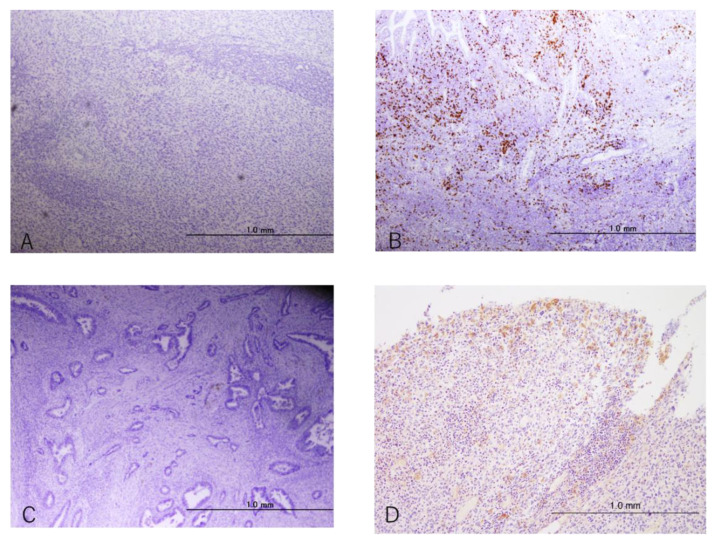
(**A**,**B**) Representative images of CD8 staining in type II EC. (**A**) Negative expression of CD8 (score 0). (**B**) Positive expression of CD8 (score 3+). (**C**,**D**) Representative images of PD-L1 staining in type II EC. (**C**) Negative expression of PD-L1. (**D**) Positive expression of PD-L1.

The relationship between MMR status, CD8+, and PD-L1 expression was assessed using a chi-square test. In the dMMR group, the positivity rate of CD8+ (*p* = 0.0072) and PD-L1 (*p* = 0.0061) expression was higher than in the pMMR group (Table 2 and Table 3, Figure 2A–D).

### 3.4. Prognostic Analysis Using the Kaplan-Meier Method

**Figure 3:**
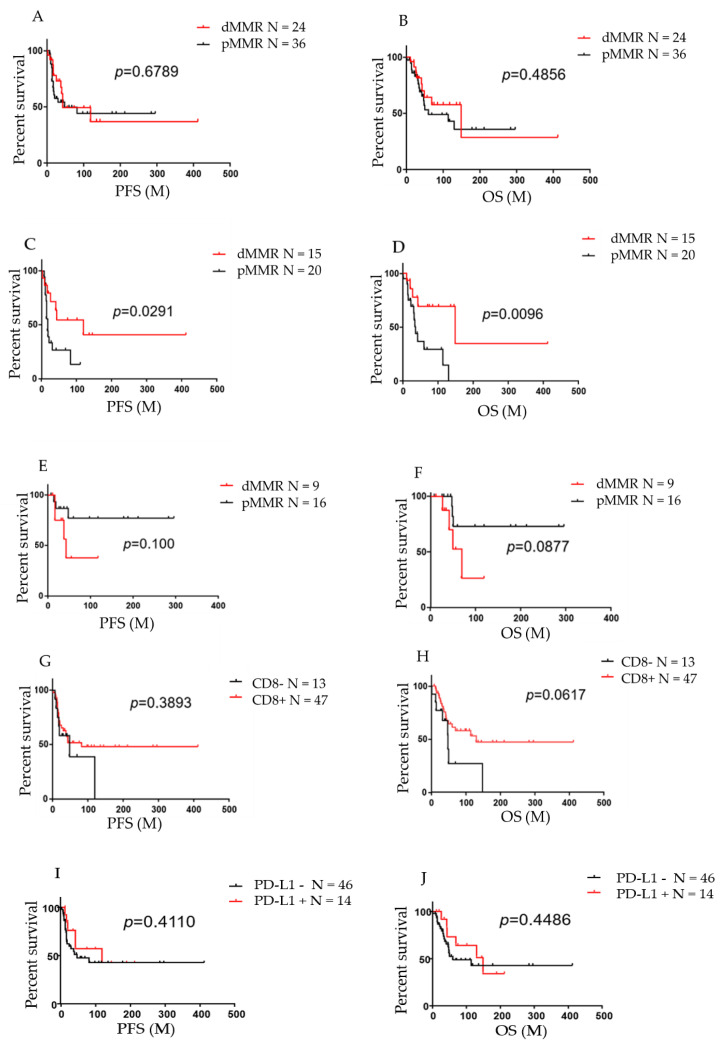
Prognostic analysis using Kaplan-Meier curves. (**A**) PFS (left panel) and (**B**) OS (right panel) analysis of type II EC patients between dMMR and pMMR group combining all stages together. (**C**) PFS (left panel) and (**D**) OS (right panel) analysis of type II EC patients between dMMR and pMMR groups for stage III/IV cases. (**E**) PFS (left panel) and (**F**) OS (right panel) analysis of type II EC patients between dMMR and pMMR groups for stage I/II cases. (**G**) PFS (left panel) and (**H**) OS (right panel) of type II EC patients with and without CD8 expression combining all stages together. (**I**) PFS (left panel) and (**J**) OS (right panel) of type II EC patients with and without PD-L1 expression combining all stages together.

Survival curves were created for the PFS and OS of patients within the dMMR and pMMR groups. We found no significant difference in PFS or OS between the dMMR and pMMR groups (Figure 3A,B), evaluated by univariate analysis within all stages. When univariate analysis was performed separately in stage I/II and stage III/IV cases for PFS or OS, in cases of stage III/IV, dMMR tumors were found to be significantly associated with longer PFS (*p* = 0.0291) and OS (*p* = 0.0096) than were pMMR tumors (Figure 3C,D). In contrast, there was no significant differences in PFS and OS between dMMR and pMMR cases in stage I/II patients (Figure 3E,F). Similarly, there were no significant differences in PFS or OS between the CD8 (+) and CD8 (−) cases (Figure 3G,H), as well as PD-L1 (+) and PD-L1 (−) cases (Figure 3I,J).

## 4. Discussion

EC is the most frequent gynecological malignancy. While the prevalence of EC is generally lower in Asian nations, it has rapidly increased in Taiwan, Korea, and Japan [41]. There are two types of EC. Type I is more common (70 to 80%) and is lower risk, estrogen-dependent, diploid, and low grade with a good prognosis; whereas type II is less common (20–30%), more aggressive, estrogen independent, less well differentiated, detected at a later stage, has a poorer prognosis, and is highly resistant to standard therapies. Therefore, type II ECs are responsible for most EC-related deaths. Type I and type II ECs differ from one another in terms of their molecular and genetic characteristics. Loss-of-function mutations in PTEN, activation of the PI3K/AKT/mTOR pathway, and MSI are more prevalent in type I ECs [42,43,44]. The most prominent genetic changes in type II ECs are p53 mutations and HER2 overexpression [44,45]. The diverse genetic mutations seen in type I and type II ECs imply that the etiologies of these subtypes may be different. While the nature of the tumors and genetic alterations are different, type II ECs are still treated in the same way as type I ECs. There are no special, targeted therapies for type II ECs. To this day, the standard treatment for all ECs is total hysterectomy and bilateral salpingo-oophorectomy with or without lymphadenectomy, followed by chemotherapy with or without radiation therapy [46]. Therefore, novel therapeutic strategies are being sought. According to data from recent clinical studies, immunotherapy with ICIs may be a potential treatment approach for people with dMMR malignancies. Among gynecological cancers, the proportion of dMMR in ovarian cancer was reported to be 2–20% [47,48,49,50,51]. We previously reported that very few dMMR cases in ovarian cancer (2.6% high-grade serous carcinoma, 7.7% mucinous carcinoma, 8.7% EC, and 4.2% clear cell carcinoma) could be effectively treated with ICI monotherapy [52]. In recent reports, the prevalence of MMR deficiency in EC is 25–30% [35,36,37]. We recently reported that the expression of CD8 and PD-L1/PD-1 was significantly higher in the dMMR group than in the pMMR group in type I EC patients. This suggests that dMMR could be used as a biomarker for ICI treatment [38]. Notwithstanding, there are currently no reports on the relationship between the MMR status and the benefit of ICIs in type II EC in the clinical setting.

In this study, MMR deficiency was observed to be 40%, which is more frequent than type I EC (28.2%) [38]. dMMR was significantly associated with immune checkpoint molecule PD-L1 expression (*p* = 0.0061) and the presence of tumor-infiltrating lymphocytes (CD8+) (*p* = 0.0072). The higher expression of CD8+ in the dMMR group confirmed the enhanced immune response. The increased level of PD-L1 in the dMMR group in this study indicates that PD-L1 promotes tumor immune escape. PD-L1 expression is closely related to dMMR/MSI-H status. Both Gatalica and Inaguma reported that the proportion of PD-L1 expression was significantly higher in dMMR/MSI-H colorectal cancer than in pMMR/MSS colorectal cancer [53,54]. Previously, it was reported that ICIs find application in cases with high infiltration of CD8+ lymphocytes and high PD-L1 expression [55,56,57,58,59,60]. Previous and present results suggest that type II EC patients in the dMMR group are good candidates for ICI treatment.

In this study, no statistical correlation was observed between the dMMR and pMMR groups in the survival curves when we performed a univariate analysis of all stages combined. However, when the univariate analysis was performed separately according to the stage I/II and III/IV, longer PFS (*p* = 0.0291) and OS (*p* = 0.0096) were observed in the dMMR group than in the pMMR group in the case of stage III/IV. In addition, although no significant difference was found but tended to have poor prognosis in dMMR group in stage I/II patients. Several reports have shown that dMMR tumors have a better prognosis than do pMMR tumors in colorectal and gastric cancer [61,62,63]. dMMR tumors have proven to be more immunogenic, have better antitumor immune responses, and be capable of inhibiting tumor cell growth. In another study, no significant difference in survival rate between the dMMR and pMMR groups was observed in EC [38] and ovarian cancer [52]. Cumulatively, the effect of the MMR status on prognosis remains controversial. In this study, dMMR showed good prognostic factors in stage III/IV cases, although no involvement was observed in stage I/II cases. The prognosis of EC in stage I/II is generally excellent, and the outcome is favorable, while stage III/IV EC is aggressive with poor outcomes and high mortality. As dMMR patients with stage III/IV showed a trend towards better prognosis, using ICIs is expected to be more effective and beneficial. Conversely, developing novel therapeutic options for patients with pMMR in stage III/IV of type II EC is imperative.

The main limitation of this study was the small population analyzed. Therefore, further experimentation using a larger population of Japanese type II EC patients is necessary. In the present study, we analyzed whether dMMR could be a biomarker for the use of ICIs in patients with type II ECs with dMMR and observed that patients with dMMR in stage I/II tended to have a poor prognosis. Therefore, the use of ICIs might be more advantageous and beneficial for such patients. Further real-world data on the effects of ICIs on type II EC with dMMR are essential.

Dostarlimab, an inhibitor of PD-1, was found to have a long-lasting effect on dMMR tumors; in 2022, a 100% remission rate was reported for rectal cancer [64]. The phase I GARNET trial assessed the safety, tolerability, and antitumor activity of dostarlimab in patients with dMMR or MSI-H recurrent or advanced EC. The results revealed significant clinical activity, durable responses, and a favorable safety profile, with no adverse effect on the quality of life [65]. The combination of dostarlimab and pembrolizumab has shown impressive results in MMR-deficient cases [66]. Recently, pembrolizumab, an ICI, was approved as a second-line treatment of metastatic or recurrent EC with MSI-H or dMMR status [67,68]. In the phase II KEYNOTE-158 trial, pembrolizumab exhibited a 57% response rate in MSI-H ECs [69]. In preclinical studies, the combination of lenvatinib and pembrolizumab was evaluated, and a synergistic antitumor activity was observed with the combination treatment more than with either treatment alone [70,71]. The Japanese Ministry of Health, Labor, and Welfare has approved this combination (pembrolizumab (Keytruda) and lenvatinib (Lenvima)) for use in patients with unresectable, advanced, or recurrent EC. The approval was based on results from the phase 3 KEYNOTE-775/study 309 trial, in which the use of pembrolizumab and lenvatinib demonstrated a statistically significant improvement in OS and PFS, reducing the risk of death and disease progression by 38% and 44%, respectively. The mean OS and PFS was 18.3 and 7.2 months, respectively [72]. Although the combination of pembrolizumab and lenvatinib was proven efficacious in EC, further clinical studies are required to confirm their safety and efficacy in type II EC. Consequently, pembrolizumab and lenvatinib therapy needs to be widely used in patients with type II EC to obtain more information about their response.

## 5. Conclusions

The high expressions of PD-L1 and CD8 positive T cells in the dMMR type II EC tumors in this study suggest that ICIs could be effective in the Japanese population; however, this should be directly tested, preferably in a large cohort, prospective study. The presence or absence of MMR proteins by immunostaining could be biomarkers for ICI response in the case of type II EC.

## Figures and Tables

**Table 1:** Relationship between MMR status and clinicopathological factors.

Characteristic	dMMR	pMMR	*p*-Value
	N = 24	N = 36	
Age-no. (%)			0.093
<60	11 (46)	9 (25)	
>60	13 (54)	27 (75)	
histological type. (%)			0.263
G3 · serous	10 (42)	10 (28)	
DDEC · CS	14 (58)	26 (72)	
FIGO Stage-no. (%)			0.593
I · II	9 (37.5)	16 (44)	
III · IV	15 (62.5)	20 (56)	
Pelvic lymph metastasis-no. (%)			0.093
No	13 (54)	27 (75)	
Yes	11 (46)	9 (25)	
Muscle invasion-no. (%)			0.733
<50	7 (29)	12 (33)	
>50	17 (71)	24 (67)	

**Table 2:** Relationship between status of MMR and CD8 expression.

Parameter	dMMR	pMMR	*p*-Value
	N = 24	N = 36	
CD8-no. (%)			0.0072
positive	23 (96)	24 (67)	
negative	1 (4)	12 (33)	

**Table 3:** Relationship between status of MMR and PD-L1 expression.

Parameter	dMMR	pMMR	*p*-Value
	N = 24	N = 36	
PD-L1-no. (%)			0.0061
positive	10 (42)	4 (11)	
negative	14 (58)	32 (89)	

## Data Availability

The data presented in this study are available on request from the corresponding author (K.N.).

## Supplementary Materials

The following supporting information can be downloaded at: https://www.mdpi.com/article/10.3390/healthcare11081073/s1, Table S1: Expression of MMR protein in type II EC patients with dMMR by IHC
